# Diagnostic Challenges during Inflammation and Cancer: Current Biomarkers and Future Perspectives in Navigating through the Minefield of Reactive versus Dysplastic and Cancerous Lesions in the Digestive System

**DOI:** 10.3390/ijms25021251

**Published:** 2024-01-19

**Authors:** Ioannis S. Pateras, Ana Igea, Ilias P. Nikas, Danai Leventakou, Nektarios I. Koufopoulos, Argyro Ioanna Ieronimaki, Anna Bergonzini, Han Suk Ryu, Antonios Chatzigeorgiou, Teresa Frisan, Christos Kittas, Ioannis G. Panayiotides

**Affiliations:** 1 2nd Department of Pathology, “Attikon” University Hospital, Medical School, National and Kapodistrian University of Athens, 124 62 Athens, Greece; danaileventakou@gmail.com (D.L.); nkoufo@med.uoa.gr (N.I.K.); anismed03@yahoo.gr (A.I.I.); ioagpan@med.uoa.gr (I.G.P.); 2 Instituto de Investigación Sanitaria de Santiago de Compostela (IDIS), 15706 Santiago de Compostela, Spain; ana.igea.fernandez@sergas.es; 3 Mobile Genomes, Centre for Research in Molecular Medicine and Chronic Diseases (CiMUS), University of Santiago de Compostela (USC), 15706 Santiago de Compostela, Spain; 4 Medical School, University of Cyprus, 2029 Nicosia, Cyprus; 5 Center for Infectious Medicine, Department of Medicine Huddinge, Karolinska Institutet, Karolinska University Hospital, Alfred Nobels Allé 8, 141 52 Stockholm, Sweden; anna.bergonzini@ki.se; 6 Department of Molecular Biology and Umeå Centre for Microbial Research (UCMR), Umeå University, 901 87 Umeå, Sweden; teresa.frisan@umu.se; 7 Department of Pathology, Seoul National University Hospital, Seoul 03080, Republic of Korea; karlnash@naver.com; 8 Department of Physiology, Medical School, National and Kapodistrian University of Athens, 115 27 Athens, Greece; achatzig@med.uoa.gr; 9 Department of Histopathology, Biomedicine Group of Health Company, 156 26 Athens, Greece; ckittas@med.uoa.gr

**Keywords:** inflammation, tissue response, reactive atypia, dysplasia, cancer, immunohistochemistry, biomarkers, molecular biology, digital pathology, artificial intelligence, pathology

## Abstract

In the setting of pronounced inflammation, changes in the epithelium may overlap with neoplasia, often rendering it impossible to establish a diagnosis with certainty in daily clinical practice. Here, we discuss the underlying molecular mechanisms driving tissue response during persistent inflammatory signaling along with the potential association with cancer in the gastrointestinal tract, pancreas, extrahepatic bile ducts, and liver. We highlight the histopathological challenges encountered in the diagnosis of chronic inflammation in routine practice and pinpoint tissue-based biomarkers that could complement morphology to differentiate reactive from dysplastic or cancerous lesions. We refer to the advantages and limitations of existing biomarkers employing immunohistochemistry and point to promising new markers, including the generation of novel antibodies targeting mutant proteins, miRNAs, and array assays. Advancements in experimental models, including mouse and 3D models, have improved our understanding of tissue response. The integration of digital pathology along with artificial intelligence may also complement routine visual inspections. Navigating through tissue responses in various chronic inflammatory contexts will help us develop novel and reliable biomarkers that will improve diagnostic decisions and ultimately patient treatment.

## 1. Introduction

“we will take inflammation, which is universally admitted to be the most important phenomenon in pathology”—E. Metchnikoff.

Inflammation comes from the Latin word inflammare meaning “to set on fire”. It is a physiological response of innate and adaptive immunity to noxious stimuli such as infection and tissue damage. Clinically, acute inflammation begins within seconds to minutes and is characterized by five cardinal signs: rubor (redness), tumor (swelling), calor (heat), and dolor (pain), identified by C. Celsus in the 1st century A.D., and functio laesa (loss of function) documented in the 19th century by R. Virchow [1]. In the late 19th and early 20th century, A.V. Waller, F.D. von Recklinghausen, J.F. Conheihm, E. Metchnikoff, and T. Lewis addressed the vascular and cellular changes in inflammation, characterized by increased vascular permeability, leukocyte recruitment, and accumulation, providing insight into the microscopic events that occur during the inflammatory response. Along this line, a key histological feature of acute response is the migration of polymorphonuclear neutrophils, which dominate the area of injury within 24 h. A successful inflammatory response is coupled with the resolution of inflammation and tissue repair. To effectively mitigate the acute inflammatory response, proinflammatory signaling pathways are counterbalanced by anti-inflammatory mediators that favor the return of homeostasis [2]. Notably, resolution programs are initiated shortly after the inflammatory response begins to prevent collateral tissue damage [2,3]. Inadequate resolution of inflammation shifts basal homeostasis to a state of persistent inflammation [4]. In chronically inflamed tissues, various immune and non-immune stromal cells engage in complex and ill-defined sustained interactions with the parenchymal cells. Key orchestrators in the choreography of chronic inflammation include transcription factors (such as Nuclear factor-kappa B (NF-κB)), cytokines, chemokines, adhesion molecules, angiogenic factors, growth factors, matrix-remodeling proteases, reactive oxygen and nitrogen species (RONS), and enzymes in the prostaglandin synthase pathway such as Cyclooxygenase 2 (COX2) [5,6]. In this context, chronicity triggers both beneficial and maladaptive tissue responses. For instance, in the context of persistent inflammatory conditions, parenchymal cells adapt to irritant stimuli by changing their identity, as exemplified by intestinal metaplasia in the esophagus or stomach, and squamous metaplasia in the lung airway [7]. Metaplasia, a Greek word meaning “to mold into a new form”, is defined as the conversion of one differentiated cell type to another, which is not normally present in a specific organ. Despite short-term benefits, such tissue adaptive responses can result in harmful long-term effects; metaplasia can be a precursor to the dysplasia–cancer sequence [7].

The first observation associating inflammation with cancer was attributed to R. Virchow, who claimed that the presence of “lymphoreticular infiltration of tumors” reflects the origin of cancer in a background of persistent inflammation [8]. Epidemiological studies have shown that chronic inflammation is associated with increased cancer incidence in the corresponding organs [6,9]. Inflammation is the denominator between cancer and common cancer-causing agents, including tobacco smoking, obesity, and environmental pollutants [10]. Moreover, certain infectious agents may establish a persistent infection within the host, which in turn promotes chronic inflammation and may induce cancer initiation [11]. An estimated 13% of all cancer cases (excluding non-melanoma skin cancers) are attributed to infectious agents [12]. Notably, increased levels of circulating inflammatory markers, such as C-reactive protein (CRP), are associated with an elevated risk of cancer in the breast, ovaries, colon, lungs, and prostate [13]. In 2011, Hanahan and Weinberg introduced the term “tumor-promoting inflammation” as an enabling characteristic, appreciating the impact of persistent inflammation on the acquisition of several cancer hallmarks by incipient cells [14]. Briefly, long-term exposure to inflammatory mediators leads to the accumulation of genetic and epigenetic changes that alter key cellular homeostatic pathways and enhance cancer development. Excellent reviews describing the underlying mechanisms of inflammation-associated carcinogenesis in detail can be found elsewhere [5,9,15,16,17].

Here, we elaborate on the tissue responses to sustained inflammatory signals in different organs and their potential association with cancer. While the morphological alterations are well described, the underlying molecular mechanisms involved in tissue adaptation remain poorly defined. We pinpoint the histopathological challenges encountered in chronic inflammatory settings and refer to tissue-based biomarkers that could help differentiate reactive atypia from true dysplasia and cancer. As, in the setting of pronounced inflammation, changes in the epithelium may overlap with neoplasia, often rendering it impossible to establish a diagnosis with certainty, deciphering the deregulation of tissue integrity in chronic inflammation offers unlimited opportunities to develop novel tissue-based biomarkers with clinical utility.

## 2. Tissue Response during Chronic Inflammation and Diagnostic Dilemmas

This section summarizes clinically significant histopathological responses in different chronic inflammatory settings, focusing on the gastrointestinal tract, pancreas, gallbladder, extrahepatic bile ducts and liver, integrating the current knowledge of the underlying molecular events. The risk of cancer in different organs is also highlighted.

### 2.1. Gastrointestinal Tract

Chronic inflammation of the mouth and esophagus stratified squamous epithelium and of the gastric and intestinal simple columnar epithelium can trigger tissue-specific adaptations.

#### 2.1.1. Mouth

**Figure 1:**
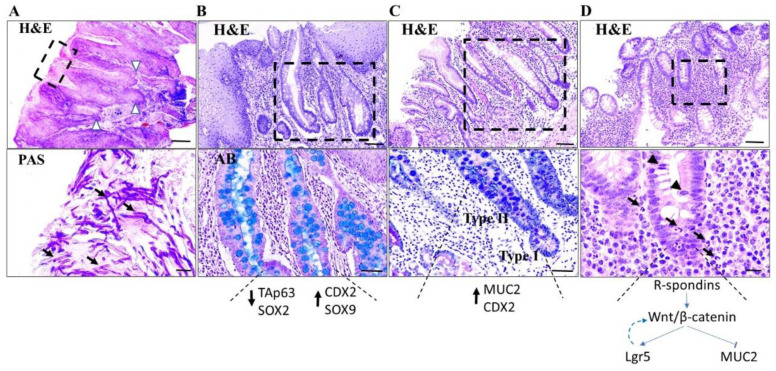
Tissue response upon chronic injury in the gastrointestinal tract. (**A**). Representative hematoxylin and eosin (H&E) staining micrograph from the oral mucosa showing pseudoepitheliomatous hyperplasia, visualized by epithelial hyperplasia along with irregular infiltrative tongue-like cords of squamous cells. The latter extend into the dermis with a pseudo-invasion pattern (arrowheads) and are accompanied by a marked inflammatory infiltrate. Periodic Acid Schiff (PAS) reaction micrograph highlights hyphae (arrows), supporting a fungal infection. Scale bar: 400 μm (H&E); 20 μm (PAS) (**B**). Representative hematoxylin and eosin (H&E) staining micrograph of esophageal mucosa with Barrett’s esophagus; notice the presence of intestinal metaplasia characterized by mucin-secreting goblet cells staining intensely blue with Alcian blue (AB). Scale bar: 100 μm (upper photo); 20 μm (lower photo). Downregulation of the squamous cell marker TAp63 and SRY (sex-determining region Y)-box 2 (SOX2), along with upregulation of the intestinal markers Caudal-type homeobox 2 (CDX2) and SRY (sex-determining region Y)-box 9 (SOX9), promote reprogramming of squamous cells into columnar epithelium (**C**). Representative hematoxylin and eosin (H&E) staining micrograph of gastric mucosa with intestinal complete (type I) (lower part) and incomplete (type II) (upper part) metaplasia; notice the presence of mucin-secreting goblet cells stained intensely blue with Alcian blue (AB). Mucin 2 (MUC2) along with CDX2 drives intestinal metaplasia phenotype. Scale bar: 100 μm (upper photo); 20 μm (lower photo). (**D**). Area indefinite for dysplasia in colonic biopsy in the setting of active inflammation due to inflammatory bowel disease. R-spondin-Wnt/β-catenin-LGR5 axis plays an essential role for the maintenance and expansion of intestinal stem crypt base cells; β-catenin transcriptionally induces Leucine-rich repeat-containing G protein-coupled receptor 5 (LGR5), while it represses MUC2 that is associated with loss of mucin. Arrows depict neutrophils; arrowheads demonstrate mitotic figures. Scale bar: 100 μm (upper photo); 20 μm (lower photo).

Pseudoepitheliomatous hyperplasia (PEH) is the reactive proliferation of epithelial cells lining the oral mucosa and epidermis in response to different irritating stimuli, including chronic inflammatory conditions [18]. Histologically, PEH in the oral mucosa is characterized by hyperkeratotic, irregular, infiltrative tongue-like cords or nests of squamous cells extending into the dermis with a pseudo-invasion pattern, often associated with inflammatory cell infiltration. Due to histopathological similarities with well-differentiated squamous cell carcinoma (SCC), the diagnosis of PEH can be challenging, especially in superficial or misoriented biopsies (Figure 1A). The histological features that favor the diagnosis of SCC include marked nuclear atypia, numerous mitoses, atypical mitotic figures, necrosis, and epithelial invasion deep into the underlying connective and muscle tissues. However, the presence of nuclear atypia and typical mitoses in PEH hampers diagnostic accuracy. On the other hand, the co-existence of inflammatory, infectious, malignant, or traumatic backgrounds favors PEH. In difficult cases, several biopsies are necessary for a definitive diagnosis.

Understanding the etiopathogenesis of PEH may help arrive at a correct diagnosis and avoid unnecessary interventions. Accumulating evidence suggests the involvement of the epidermal growth factor receptor (EGFR)–epidermal growth factor (EGF) axis, fibroblast growth factor 7 (FGF7), stem cell factor–c-kit receptor axis, transcription growth factor α (TGFα), transcription growth factor β1 (TGFβ1), and T helper type I cytokines, namely interferon γ (INFγ) and TNFα (tumor necrosis factor α) through autocrine and paracrine pathways in PEH pathogenesis [19,20,21]. A unique molecular signature has been identified in PEH and cutaneous SCC, including 703 differentially expressed genes between the two entities [22]. Interestingly, the most significant differences were found in metabolic pathways, including oxidative phosphorylation and polyamine biosynthesis, providing insight into the pathogenesis of PEH and SCC, which may aid in differential diagnosis and serve as potential targets for therapeutic interventions.

#### 2.1.2. Esophagus

Chronic gastroesophageal reflux of gastric acid and bile leads to mucosal injury associated with inflammation, creating a permissive environment for intestinal metaplasia, also known as Barrett’s esophagus (BE). The latter is determined by the replacement of differentiated squamous epithelial cells with columnar epithelium and goblet cells, as part of the wound-healing process (Figure 1B) [7]. Experimental data show that the glandular epithelium has a proliferative advantage over squamous epithelium in an acidic environment, arguing for the short-term benefits of this tissue response [23].

The potential origins for metaplasia in BE are the basal cells of squamous epithelium, residual embryonic cells, transitional basal cells at the gastro-esophageal junction, gastric gland cells and the esophageal submucosal glands [23]. To date, there has been a lack of experimental models to prove the source of esophageal metaplasia. Mechanistically, accumulating evidence demonstrates that repeated injury of the esophageal epithelium deregulates key transcription factors implicated in esophageal embryogenesis. Downregulation of the squamous cell marker TAp63, a p53 human homolog of p63, retains the N-terminal activation domain, and SRY (sex-determining region Y)-box 2 (SOX2), along with upregulation of the intestinal markers Caudal-type homeobox 2 (CDX2) and SRY (sex-determining region Y)-box 9 (SOX9), favors the reprogramming of squamous cells into the columnar epithelium [24] (Figure 1B). BE is a major risk factor for esophageal adenocarcinoma; the annual risk for esophageal cancer increases up to 6% in patients with BE who develop high-grade dysplasia [25].

#### 2.1.3. Stomach

Prolonged inflammation in the stomach (also known as chronic gastritis) is triggered by environmental (such as *Helicobacter pylori* infection) or autoimmune damage of the gastric mucosa. Failure of the injured gastric glands to regenerate progressively leads to fibrosis, resulting in gastric atrophy. Within this context, certain metaplastic changes can occur, including intestinal, pyloric, pseudo-pyloric, and pancreatic acinar metaplasia [26]. Intestinal metaplasia refers to the replacement of the gastric mucosa by small bowel epithelium with a brush border (complete (type I)) or the large bowel epithelium without a brush border (incomplete (type II)) (Figure 1C). Type I intestinal metaplasia is characterized by gain of intestinal type mucin 2 (MUC2) expression and absence or decreased expression of gastric-related mucins mucin 1 (MUC1), mucin 5AC (MUC5AC), and mucin 6 (MUC6) [27]. On the other hand, in type II intestinal metaplasia, MUC2 is co-expressed with the gastric-related mucins (Figure 1C). Concerning gastric intestinal metaplasia pathogenesis, the causative role of intestinal transcription factors caudal-type homeobox 1 (CDX1) and CDX2 has been appreciated [28] (Figure 1C). Interestingly, intestinal metaplasia is stable even after resolution of inflammation [27]. According to the Correa model, gastric atrophy and intestinal metaplasia are precursors of gastric adenocarcinoma; therefore, it is important to detect these lesions to identify at-risk patients [29]. The presence of incomplete intestinal metaplasia increases the cancer risk [30]. In pyloric metaplasia, the normally occurring fundic-type glands are replaced by mucus-secreting glands expressing MUC6, while they are negative for pepsinogen I, which is normally expressed by chief cells in the gastric oxyntic region. In pseudo-pyloric metaplasia, the metaplastic glands stain positive for both MUC6 and pepsinogen I [31]. Studies in animal models of acute parietal cell loss have revealed another type of metaplasia known as spasmolytic polypeptide-expressing metaplasia (SPEM). During parietal cell loss, IL-33 is released by foveolar epithelial cells and stromal cells, including alternatively activated macrophages (also known as M2), upregulating type II cytokines (including IL-4 and IL-13), which in turn favors SPEM [32]. Transdifferentiation of chief cells into SPEMs is associated with upregulation of trefoil factor family 2 (TFF2) and CD44 variant 9 (CD44v9) [32]. During SPEM, the expression of genes related to the secretory phenotype (such as secreting digestive enzymes) is scaled down, while genes related to wound repair are scaled up [33]. This process exhibits striking similarities with acinar-to-ductal pancreatic metaplasia (ADM, analyzed below). In humans, SPEM is found in the fundus of *Helicobacter pylori* related gastritis and in the mucosa adjacent to dysplasia-carcinoma areas [32,34]. Pancreatic acinar metaplasia is found in less than 1% of patients with chronic gastritis and is often associated with *Helicobacter pylori* infection [35]. It is more frequent in the antrum than in the corpus and comprises clusters of pancreatic acinar cell-like cells, along with exocrine cells with basophilic cytoplasm that are positive for B-cell lymphoma/leukemia (BCL-10) and α-amylase. There is no conclusive evidence linking pancreatic acinar metaplasia with cancer risk.

#### 2.1.4. Colon

Chronic and relapsing mucosal tissue damage followed by wound healing is a key feature of Inflammatory bowel disease (IBD), which presents as Crohn’s colitis (CC) and ulcerative colitis (UC) [36]. Severe intestinal inflammation leads to epithelial loss and degradation of the extracellular matrix, which is clinically evident as erosions or ulcers. Tissue regeneration is compromised by recurrent damage. Consistent morphological markers of chronic injury include crypt architectural distortion, basal plasmacytosis, diffused mixed lymphoplasmacytic infiltrate within the lamina propria, basal lymphoid aggregates, pyloric metaplasia, distal Paneth cell metaplasia and fibrosis [37]. Crypt architectural distortion which is frequently observed, is considered a hallmark of chronic injury, and reflects the presence of ongoing inflammation or regeneration with neo-formation of crypts. It is characterized by irregularly arranged, branched, dilated, or shortened crypts, such as L-shaped or T-inverted crypts, often adjacent to colonic ulcers [38].

During intestinal inflammation and tissue damage, quiescent stem cells residing at the bottom of the crypt, along with progenitor and terminally differentiated cells that re-enter the cell cycle promote intestinal [39]. Mechanistically, the secretion of inflammatory mediators, including TNFα, induces Wingless-related integration site (Wnt)/β-catenin signaling, which in turn favors mucosal healing [40,41]. The Wnt/β-catenin pathway is a key regulator of intestinal homeostasis, regulating the stem cell compartment and favoring maintenance of the proliferating zone [42]. Intestinal stem cells residing in the bottom crypt express Leukine-rich repeat-containing G protein-coupled receptor 5 (Lgr5), a receptor for a family of Wnt agonists called R-spondins secreted by mesenchymal and Paneth cells. Lgr5 is also a direct downstream target of Wnt/β-catenin signaling [41,43]. Collectively, the R-spondin-Wnt/β-catenin-LGR5 axis is essential for the maintenance and expansion of intestinal stem crypt base cells; pathway inhibition leads to the loss of Lgr5+ crypt base columnar cells, while R-spondin administration improves tissue regeneration [41,42] (Figure 1D). Interestingly, evidence supports that Lgr5 potentiates Wnt/β-catenin signaling forming a positive loop [44]. Moreover, β-catenin signaling downregulates MUC-2, an abundantly expressed mucoprotein produced by goblet cells in the intestine [45] (Figure 1D). The latter is in line with the fact that in active UC, goblet cells are reduced, and the remaining goblet cells cannot efficiently synthesize MUC-2 [46], which is associated with defective mucus secretion and barrier formation.

Gut fibrosis is a common complication of repetitive tissue injury in IBD. Fibrosis accounts for approximately 50% of Crohn’s disease and less than 11% of UC cases [47]. Cytokines, growth factors, and intestinal microorganisms activate myofibroblasts, thereby enhancing intestinal fibrosis. During intestinal fibrosis, the equilibrium between anti-inflammatory matrix metalloproteinases (MMPs) and tissue inhibitors of MMPs (TIMPs) is deregulated. Increased expression of TIMP1 along with upregulation of fibroblast activation protein (FAP), plasminogen activator inhibitor 1 (PAI-1), and Cadherin-11 favors the fibrotic process [47].

Anatomical extent, duration of colitis, and severity of inflammation are well-established risk factors for colorectal cancer development in patients with IBD [48]. Colitis-associated cancer (CAC) develops through a chronic inflammation–dysplasia–carcinoma sequence [49]. Importantly, the differentiation of reactive dysplasia from true dysplasia in the setting of chronic active inflammation can be challenging. Regenerating intestinal epithelium often shows mucin loss, enlarged hyperchromatic nuclei with prominent nucleoli, nuclear stratification, and increased mitotic figures, mimicking true dysplasia (Figure 1D). Epithelial surface maturation and lack of atypical mitotic figures in the setting of active inflammation favors the diagnosis of reactive lesion. Interestingly, gross genomic alterations, such as DNA aneuploidy, can be widespread in the intestinal mucosa in the absence of dysplasia, identifying a subset of IBD patients that require more intense surveillance [50]. When a definite diagnosis cannot be established with certainty, cases are classified as “indefinite for dysplasia” [51]. Notably, in a retrospective analysis, IBD patients with mucosal changes classified as indefinite for dysplasia had an increased risk of CAC, underscoring the importance of colorectal neoplasia surveillance [51].

### 2.2. Pancreas

The pancreas is a gland that includes both exocrine and endocrine components, and mainly consists of epithelial elements, that is, acini, ducts, and islets of Langerhans, with minimal intralobular stroma. Chronic pancreatitis (CP) is a fibroinflammatory disorder characterized by progressive fibrotic destruction of the pancreatic parenchyma, leading to exocrine and endocrine insufficiency [52]. Fibrosis, atrophy, and duct changes are hallmarks of CP; however, there are no specific histological features to distinguish the different etiologies of CP [53]. Interlobular and intralobular fibrosis accompanied by acinar loss, distortion, and dilatation of ducts, along with calcification and pseudocysts (cavities lacking an epithelial lining), are histological characteristics of CP. Although lymphocytic aggregates may be present, the inflammatory infiltrate is scant.

The exact mechanism underlying CP pathogenesis is not well understood. It has been postulated that chronic injury leads to cell death and subsequent release of cytokines, including fibrogenic platelet-derived growth factor (PDGF) and TGFβ1, which in turn activate the pancreatic stellate cells (PSCs) [54]. Quiescent PSCs are resident cells of the pancreas that contain retinoid lipid droplets, express vimentin and glial fibrillary acidic protein (GFAP), and possess stem cell/progenitor features [54]. Activated PSCs acquire features of myofibroblast-like cells; they express α smooth muscle antigen (αSMA), produce extracellular matrix such as collagen type I and III, laminin, and fibronectin, and secrete cytokines. The latter promotes the recruitment of additional inflammatory cells, fueling a feed-forward loop of pancreatitis identified by a stiff fibrotic tissue. Other factors, such as alcohol consumption, oxidative stress, and hypoxic conditions can directly activate PSCs [55]. Accumulating evidence suggests that activation of the mitogen-activated protein kinase (MAPK) signaling pathway in PSCs promotes proinflammatory cytokine production, fibrosis, and ADM (analyzed below). On the other hand, systemic inhibition of MAPK signaling attenuates fibrosis and inflammation while compromising tissue regeneration [56]. Interestingly, treatment with the Peroxisome proliferator-activated receptor-γ (PPAR-γ) ligand troglitazone inhibited PSC activation, suggesting that PPAR-γ signaling can be utilized therapeutically in CP [57].

**Figure 2:**
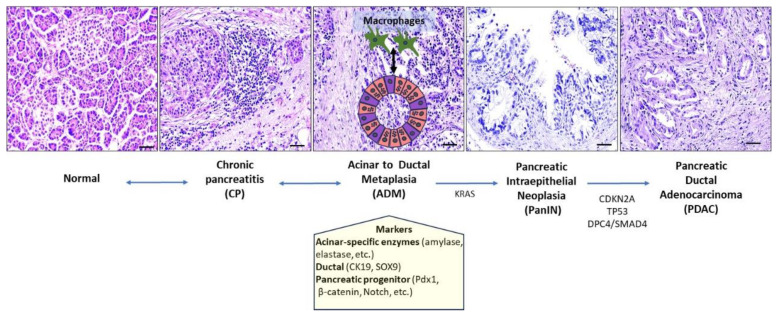
Chronic inflammation, tissue response and pancreatic ductal adenocarcinoma development. During chronic pancreatitis (CP), acinar cells may undergo acinar-to-ductal metaplasia (ADM) that is composed both of duct-like and acinar-like cells with embryonic progenitor cell properties. ADM cells stain with ductal ((Cytokeratin 19(CK19) and SRY-Box Transcription factor 9 (SOX9)), acinar (i.e., enzymes including amylase, elastase, etc.), and pancreatic progenitor ((pancreatic and duodenal homeobox 1 (Pdx1), β-catenin, and Notch)) markers. ADM is a reversible process. Upon oncogenic KRAS activation, ADM can progress towards pancreatic intraepithelial neoplastic lesion (PanIN). Macrophages have been shown to drive ADM and play a role in ADM to PanIN transition. Progression during higher-grade PanIN and pancreatic ductal adenocarcinoma (PDAC) is associated with mutations and/or allelic loss of Cyclin-Dependent kinase inhibitor A (*CDKN2A*), *TP53,* and Deleted in Pancreatic Cancer 4 (*DPC4*, also known as *SMAD4*) genes encoding the tumor suppressors P16^INK4A^, P14^ARF^, P53, and the transforming growth factor β (TGFβ) signal transducer SMAD4, respectively. Scale bar: 50 μm.

During chronic pancreatic injury, acinar cells may undergo ADM [53,58]. Acinar cells appear to be more sensitive to irritating stimuli than other pancreatic cell lineages, suggesting that ADM represents an adaptive tissue response to CP [59]. Experimental evidence has demonstrated that during ADM, acinar cells revert to a less differentiated and more proliferating state, giving rise to duct-like cells. Morphologically, the ADM structure contains both acinar-like and duct-like cells that retain cell polarity and co-express acinar-specific digestive enzymes (such as amylase elastase and trypsin) and duct markers including mucin, cytokeratin 19 (CK19), and SOX9 [60]. Furthermore, the pancreatic progenitor markers pancreatic and duodenal homeobox 1 (Pdx1), β-catenin, and Notch are upregulated in ADM [59] (Figure 2). Therefore, the term metaplasia may be misleading because there are no mature duct structures. Hence, ADM cannot be considered a pure trans-differentiation event, as it is also accompanied by a dedifferentiated phenotype. To this end, Willet et al. [61] introduced the term paligenosis (originating from the Greek *pali* (again), *gen* (birth), and *osis* (process)) to describe this process of reversion from a differentiated to a plastic cell state with cell cycle re-entry that may give rise to metaplasia. Notably, the authors demonstrated parallels between SPEM (occurring in the stomach) and ADM, suggesting that this process can be conserved across different organs, favoring tissue repair [61]. From a molecular perspective, upon damaging insult, pancreatic acinar cells decrease their metabolic activity by reducing mammalian target of rapamycin complex 1 (mTORC1) while increasing autophagic machinery, resulting in a less differentiated state that favors the expression of embryonic/wound-healing genes such as CD44v and SOX9 [33]. At the onset of ADM, activating transcription factor 3 (ATF3) promotes autodegradation in a RAB7B-dependent manner. At the same time, Basic Helix–Loop–Helix Family Member A15 (BHA15, widely known as MIST1), a key regulator of secretory cell architecture, is downregulated, explaining the downscaling of secretion. In addition, the expression of acinar-associated pancreatic transcription factor 1 subunit α (PTF1α) is also decreased [62]. At a later stage, cells reactivate their metabolism, shut down the autophagic process, and re-enter the cell cycle [33]. The inhibition of autophagy and lysosomal activity fails to downscale differentiation [33]. Similarly, administration of the mTORC1 inhibitor rapamycin leads to a loss of the capacity to proliferate while retaining the expression of metaplastic genes [33]. ADM is a reversible process; however, in response to oncogenic signaling, ADM progresses to pancreatic intraepithelial lesion (PanIN), a common precursor of pancreatic ductal adenocarcinoma (PDAC) [59] (Figure 2). Activation of TGF-β signaling, a key pathway involved in the pathogenesis of CP, in pancreatic acinar cells induces ADM and accelerates KRAS^G12D^ mediated pancreatic carcinogenesis [63]. Mechanistically, in a mouse model of CP, infiltrating macrophages with a classical activated phenotype (also known as M1) promote ADM in an NF-κB/MMP–dependent manner [64]. The release of IL-13 by ADM switches macrophage polarization from M1 to M2, which in turn promotes PanIN development in the presence of oncogenic RAS [65] (Figure 2). In humans, the juxtaposition of ADM with PanINs harboring the same KRAS mutations further supports this link [59].

One of the major diagnostic challenges in pathology is the differentiation of CP from PDAC [66]. In CP, the irregular contour of ducts, lined by epithelium exhibiting nuclear atypia within dense fibrotic tissue, can generate a diagnostic pitfall. Maintenance of lobular architecture, regardless of cellular atypia, favors benign diagnosis [67]. On the other hand, the presence of ducts adjacent to arteries, vascular and perineural invasion, and ducts suspended in peripancreatic fat are diagnostic features of carcinoma [67]. Ductal cells in PDAC often have denser eosinophilic cytoplasm than those in benign lesions. In addition, anisonucleosis (variation in cell nuclei of more than four to one within a gland) and bizarre nuclei, along with irregular nuclear contours, are considered highly suspicious for PDAC [67]. Importantly, CP is an established risk factor for PDAC [17]. The cumulative risk of PDAC is 1.8% and 4.0% at 10 and 20 years, respectively, after CP [68]. Given that ADM is a precursor for the development of PanIN [59], it is clear that chronic pancreatic injury plays a key role in the decisive steps during pancreatic carcinogenesis.

### 2.3. Gallbladder and Extrahepatic Bile Ducts

The gallbladder, the extrahepatic biliary ducts (EHBDs), the liver, and the pancreas all develop from an outpouching of the endodermal lining of the foregut called the hepatic diverticulum. SOX17, along with Pdx1, plays a decisive role in whether Pdx1+ cells are differentiated towards the pancreas or EHBD; SOX17 expression promotes biliary tract formation, while SOX17 haploinsufficiency in mice leads to gallbladder and EHBD hypoplasia [69].

The gallbladder is among the most common surgical specimens in routine practice. It is associated with the EHBD via the cystic duct. Similar to other organs of the gastrointestinal tract, prolonged injury to the gallbladder mucosa triggers metaplastic changes, pyloric (antral type) metaplasia being the most common and intestinal metaplasia occurring less frequently [70]. The glands in pyloric metaplasia are similar to gastric glands in the antrum, while glands in intestinal metaplasia contain goblet and absorptive cells with brush border reminiscent of incomplete metaplasia. Notably, in a large cohort study involving 400 surgically removed gallbladders, a significant association was found between dysplasia and intestinal metaplasia and between pyloric metaplasia and intestinal metaplasia [71]. The same study also demonstrated an age-dependent occurrence of these changes, with pyloric metaplasia occurring more frequently in younger patients, intestinal metaplasia in intermediate mean age, and dysplasia in older patients. Hence, intestinal metaplasia is believed to be more closely associated with the dysplasia–carcinoma sequence [72]

Nontumoral intraepithelial neoplasms in the gallbladder are microscopic forms of dysplasia; essentially, these are the counterparts of PanIN (described above) and biliary intraepithelial neoplasia (BillN, described below). Differentiating dysplasia from reactive atypia is challenging in gallbladder pathology, as mild nuclear atypia is common in cholecystitis. Given that molecular findings characterizing the neoplastic lesions are limited, dysplasia is distinguished from reactive lesions mainly based on morphological features. Specifically, certain architectural patterns like tall (micro)papillary and cribriform configuration favor dysplasia. Moreover, nuclear enlargement, nuclear hyperchromasia, prominent nucleoli and loss of polarity are characteristic features of dysplasia, whereas surface maturation and intraepithelial neutrophils along with ulceration and/or acute inflammation favor reactive changes [73]. Notably, the sharp demarcation of dysplastic epithelium from adjacent normal epithelium is very helpful in distinguishing dysplasia from reactive changes [71]. Rokitansky–Aschoff sinuses with reactive atypia may mimic adenocarcinoma; Rokitansky–Aschoff sinuses are perpendicular to surface and may contain luminal bile, whereas cancerous glands are arranged in a haphazard manner or are orientated parallel to the surface, may be adjacent to muscular vessels, and are associated with desmoplastic reaction [74]. Importantly, dense fibrosis surrounding Rokitansky–Aschoff sinuses is common in chronic cholecystitis and should not be confused with malignant desmoplasia [74].

The association between persistent inflammation and cancer is exemplified in the bile ducts, as chronic inflammation promotes the BillN–cholangiocarcinoma sequence [75]. Mechanistically, inflammatory mediators including Interleukin 6 (IL-6), Tumor Necrosis Factor alpha (TNFα), and COX2 induce genetic and epigenetic alterations that favor cholangiocarcinogenesis [76]. IL-6 alters the promoter methylation of several growth-associated genes, leading to increased expression of EGFR [77]; moreover, it downregulates a group of miRNAs that in turn favor the upregulation of DNA methyltransferase-1 (DNMT-1), resulting in the decreased expression of tumor suppressor genes like p16INK4A [78]. TNFα stimulates in an NF-κΒ-dependent manner the upregulation of the DNA/RNA editing enzyme activation-induced cytidine deaminase (AID) that has a mutagenic activity by converting cytosine to uracil [79]. The latter leads to the generation of somatic mutations in key genes related to cancer progression like *TP53*, *c-myc,* and the promoter region of INK4A [79]. High COX-2 promotes tumor growth, whereas COX-2 inhibition promotes apoptosis and inhibits proliferation in cholangiocarcinoma [80,81].

Distinguishing reactive atypia from BillN and adenocarcinoma in EHBD is challenging as neoplastic cells can appear deceptively benign. Moreover, in areas with active inflammation, a diagnosis of BillN is difficult, as non-neoplastic epithelium may exhibit substantial nuclear changes like hyperchromasia and enlargement [74]. Overall, nuclear enlargement, nuclear hyperchromasia, loss of polarity, and nuclear stratification favor BillN. It is rather unusual for reactive lesions to exhibit all these features [74]. Additionally, maturation towards the surface along with the presence of intraepithelial neutrophils as well as fine and pale chromatin favor reactive atypia [82]. Moreover, reactive changes lack a sharp demarcation from the surrounding adjacent epithelium. On the contrary, the cribriform pattern along with nuclear irregularity favors BillN. The mutation of the *KRAS* codon 12 is an early event, while aberrant P53 expression is a late event during the progression to BillN [83]. An increasing number of data highlight the role of autophagy, demonstrating the increased expression of autophagy-related proteins early during carcinogenesis at the BillN step [84,85]. However, additional studies are needed to understand the underlying molecular events driving the BillN–cholangiocarcinoma carcinoma sequence, which, in turn, could help us to better distinguish reactive changes from true epithelial dysplasia.

### 2.4. Liver

Hepatocytes are the liver parenchymal cells. They are arranged in anastomosing cords, separated by vascular sinusoids that link the portal triad (portal tract) with the central vein and are supported by the biliary epithelium in the canals of Hering. The hepatocytes are organized into functional units; the most relevant ones for histopathological assessment are the hepatic lobule (also known as classic) and hepatic acini. The hepatic lobules are roughly hexagonal in shape, consisting of a central vein with cords of hepatocytes radiating to portal triads set at the angles of the hexagon [86]. The acinar model defined by A. Rappaport is as an elliptical area in which blood flowing from the portal venule and hepatic arteriole drains through the liver sinusoids and empties into the terminal hepatic venule (i.e., central vein) [86]. Periportal hepatocytes are the most oxygenated, designated as zone 1; oxygenation is reduced in the intermediary zone 2 and reaches its lowest in the centrilobular zone 3, including hepatocytes around the terminal hepatic venules, which are more susceptible to ischemia and toxic-induced injury. Histologic injury is manifested as alterations in the liver architecture along with inflammation, steatosis, fibrosis, lobular injury, and ductular reactions. Chronic hepatitis is a necro-inflammatory liver disease characterized by portal, interface (periportal) and lobular inflammation, as well as necrosis and fibrosis [87]. These histopathological features are seen irrespective of the etiology.

In this review, we focus on the tissue response during nonalcoholic fatty liver disease (NAFLD), which is the most common chronic liver disease affecting 10–24% of the global population [88]. NAFLD encompasses a range of manifestations from simple steatosis to nonalcoholic steatohepatitis (NASH), advanced fibrosis, cirrhosis, and cancer [88]. Hepatic steatosis is the result of the accumulation of lipid droplets within the cytoplasm of hepatocytes. The simple form of NAFLD is defined by at least 5% hepatic steatosis [89]. In approximately one third of patients, the addition to steatosis of parenchymal tissue damage and inflammation (mainly lobular and/or portal) along with a variable degree of fibrosis results in NASH, which can potentially progress to cirrhosis and hepatocellular carcinoma (HCC) [89]. Steatosis and liver damage begin in zone 3 and with progression extending along the entire hepatic lobule [87]. Hepatocellular injury is characterized by ballooning, apoptosis, and lytic necrosis. Hepatocyte ballooning is a histopathological hallmark in NASH. It is an ill-defined form of hepatocytic injury characterized at conventional hematoxylin–eosin staining by a rounded 2–3-fold cellular enlargement with rarefied cytoplasm, often including Mallory–Denk bodies (MDBs) [90]. MDBs were described by F.B. Mallory and H. Denk; they are cytoplasmic hyaline inclusions composed of various misfolded and cross-linked proteins including cytokeratin (CK) 8 and CK18, chaperones like heat shock protein 70 (Hsp70), and components of protein degradation machinery (i.e., ubiquitin, p62) [91]. Along this line, autophagy activation by rapamycin promotes the resolution of preformed MDBs and prevents the formation of new MDBs in mice, stressing out the role of proteasomal degradation and autophagy machinery in MDB formation in NAFLD [92]. What remains unclear is whether MDBs are inert inclusions representing an epiphenomenon of chronic injury or actively contribute to NAFLD pathogenesis, exerting a protective or harmful mechanism. Interestingly, balloon cells exhibit decreased CK18 immunostaining, confirming cytoskeletal damage, whereas MDBs are positive for CK18 and p62 [90].

Currently, a multi-hit parallel model that comprises insulin resistance (IR), obesity, genetic predisposition, inflammation, oxidative stress, endoplasmic reticulum (ER) stress, and the gut microbiota reflects our knowledge of NAFLD pathogenesis [93]. The impairment of insulin signaling is a very early event in NAFLD development. In the context of IR, there is excessive hepatic fat accumulation, which overwhelms physiologically adaptive responses, leading to oxidative and ER stress that, in turn, leads to hepatocellular injury, collectively called lipotoxicity [89,94]. The lipotoxicity of hepatocytes is fundamental in the pathogenesis of NASH and is associated with inflammatory recruitment. For instance, lipid accumulation within hepatocytes activates stress-responsive C-Jun N-terminal kinase (JNK), which in turn produces proinflammatory cytokines [95]. Along this line, the excess uptake of cholesterol by Kupffer cells triggers an inflammatory response by the latter [96]. Notably, oxidative stress byproducts (i.e., oxidized lipids) act as damage-associated molecular patterns (DAMPs) that activate Toll-like receptor signaling, triggering an innate immune response [89]. Liver resident Kupffer cells, bone-marrow-derived macrophages, neutrophils, and dendritic cells are the key innate immune subpopulations in NASH [97,98]. Moreover, a key histological feature of NASH is the lobular infiltration by T and B lymphocytes. To this end, products of peroxidation not only act as DAMPs but also form epitopes known as oxidation-specific epitopes (OSEs), which trigger adaptive immunity and anti-OSE IgGs [89]. Liver inflammation is also associated with fibrosis. The production of cytokines like TNFα and Transforming Growth Factor β (TGFβ) by immune cells, including Kupffer cells and parenchymal cells, activates the hepatic stellate cells (HSCs) [99]. Quiescent HSC (also known as Ito cells) are located in the Space of Dissè and store Vitamin A. High-throughput analysis has revealed significant similarities between HSCs’ and PSCs’ features [100]. Activated HSCs become proliferating and fibrogenic αSMA(+) myofibroblasts, which in turn drive hepatic fibrosis. Interestingly, clinical models have demonstrated that the clearance of HSCs has a therapeutic benefit favoring the resolution of fibrosis. This can be mediated through the following mechanisms: (i) reversion; the deactivation of HSCs to a state similar to quiescence with a downregulation of the expression of fibrogenetic genes, (ii) apoptosis, which contributes to decreased numbers of HSCs; and (iii) senescence, which promotes immune clearance through the upregulation of genes related to immune surveillance by senescent HSCs [99,101]. To this end, an increasing number of data suggest that senescence is involved in NAFLD pathogenesis and progression to NASH [102]. Hepatic senescence promotes liver steatosis, whereas targeting senescent cells reduces steatosis, opening new therapy perspectives [103].

The risk of hepatocellular carcinoma is a growing concern in both cirrhotic and non-cirrhotic NAFLD patients. In a large prospective study, nearly 10% of cirrhotic NASH patients developed hepatocellular carcinoma; however, the risk is lower than for hepatitis C virus-associated cirrhosis [104]. Importantly, since obesity and type 2 diabetes mellitus, two established risk factors for cancer, co-exist with NAFLD, assessing the neoplastic potential of NAFLD can be challenging [89].

## 3. Tissue-Based Biomarkers Differentiating Reactive from Neoplastic Lesions

To differentiate reactive from neoplastic lesions in the context of chronic inflammation, morphological features are often complemented with immunohistochemistry (IHC), which is a cheap, quick, and easily applicable method. Table 1 [19,105,106,107,108,109,110,111,112,113,114,115,116,117,118,119,120,121,122,123,124,125,126,127,128,129,130,131,132,133,134,135,136,137,138,139,140,141,142,143,144,145,146,147,148,149,150,151,152,153,154,155,156,157,158,159,160,161,162,163,164,165,166,167,168,169,170,171,172,173,174,175,176,177,178,179,180,181,182,183,184,185,186,187,188,189,190,191,192,193,194,195] includes tissue-based biomarkers that can be useful for differentiating reactive atypia from neoplasia. For reasons of completeness, the biomarkers listed in Table 1 extend beyond the gastrointestinal tract, pancreas, gallbladder, extrahepatic bile ducts and liver and include various organs.

As shown in Table 1, the tumor suppressor p53 is often assessed using IHC to differentiate reactive atypia from neoplasia. Mutations in *TP53* (encoding p53) and the chromosomal loss of 17p, where *TP53* resides, are among the most common genetic defects documented in cancer [196]. Notably, p53 mutations often occur in the early phases of carcinogenesis [196], as exemplified by *TP53* missense mutations in dysplastic Barrett’s mucosa [190]. *TP53* non-synonymous mutations occur at a high frequency in patients with noncancerous inflamed gastric mucosa exhibiting intestinal metaplasia [197] and in colon tissue from patients with UC [198], suggesting that irreversible genetic alterations occur very early in inflammation-associated carcinogenesis. Notably, an accumulation of mutant p53 early in inflamed colonic tissues, through gain of function, acquires a proinflammatory activity in an NF-κΒ-dependent manner, which in turn promotes cancer [199]. Therefore, the evaluation of p53 status is implemented as a sensor of oncogenic transformation. Given the good correlation between IHC patterns and the presence of p53 mutations, p53 immunostaining is applicable in routine practice. For the interpretation of p53 immunostaining, the following should be taken into consideration: (a) wild-type p53 has a very short half-life, and its presence in routine practice is often below sensitivity, resulting in a mixture of negative, faint, and intense immunostaining; (b) missense mutations in *TP53* often prolong the half-life of p53, resulting in protein nuclear accumulation that, in turn, allows its detection by diffuse and intense nuclear immunostaining; and (c) homozygous deletions or truncating mutations are associated with negative p53 immunostaining, which may provide an explanation for the discrepancies between IHC and sequencing [200]. To make things more complicated, in some tumors (including melanoma and astrocytoma), there is a nuclear accumulation of p53 without overt mutations in *TP53* [201,202]. Integrating our experience from routine practice, rare cases of common cancers exhibiting intense and diffused p53 immunostaining turned out to be wild-type after sequencing. The immunohistochemistry of downstream p53 targets, such as p21^WAF1^, can improve accuracy. Wild-type p53 promotes the transcription of several downstream target genes; however, some forms of mutant p53 may also induce p21^WAF1^ expression [125]. Additionally, p21^WAF1^ can be induced in a p53-independent manner, adding to the complexity of this topic [125].

**Figure 3:**
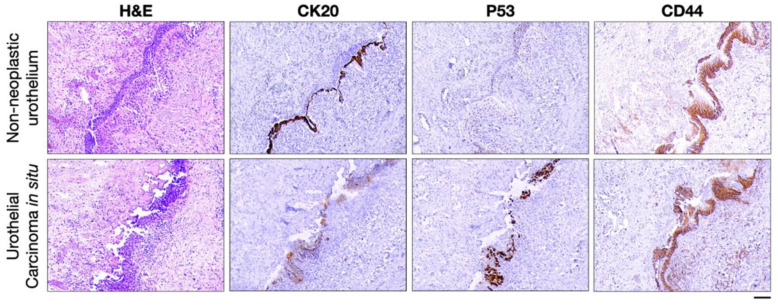
Panel of immunohistochemical markers in the differential diagnosis between reactive urothelium and urothelial carcinoma in situ. Representative hematoxylin and eosin (H&E) staining and immunohistochemistry micrographs showing CK20 expression limited to the umbrella cells, faint and patchy nuclear p53, and full-thickness CD44 immunostaining in non-neoplastic urothelium (upper photos), in contrast to full-thickness CK20, intense and diffused nuclear p53, and CD44 basal expression in urothelial carcinoma in situ (lower photos). Scale bar: 100 μm.

The proliferating marker Ki67 is commonly used to differentiate reactive lesions from dysplastic lesions (Table 1). In principle, Ki67 expression is limited to the proliferating zone, whereas in dysplastic lesions, Ki67 immunostaining is often expanded beyond the proliferating area. However, being a proliferating marker, Ki67 can be overexpressed in benign inflamed tissues undergoing tissue repair [203]. For instance, Ki67 has poor discriminating value for reactive urothelial atypia versus urothelial carcinoma in situ [148]. Hence, Ki67 reactivity alone is not reliable and is often complemented with additional markers in routine practice. For instance, in the uterine cervix, Ki67 expression was examined along with the status of the cell cycle inhibitor p16^INK4a^ to differentiate reactive atypia from dysplasia (Table 1). In addition, Maspin, Insulin-like growth factor II messenger ribonucleic acid (mRNA)-binding protein 3 (IMP3), and S100P improve sensitivity and specificity in differentiating pancreatic ductal adenocarcinoma from chronic pancreatitis (Table 1). IMP3, an RNA-binding protein involved in RNA processing, is believed to play an important role in cell growth and migration [204]. IMP3 is an oncofetal protein expressed in developing organs but is almost silenced in adult tissues, whereas it is diffusely re-expressed in malignant tissues, contributing to tumor progression [205]. An increasing number of data emphasize its role as a potential biomarker for differentiating benign from malignant lesions in different organs (Table 1) [206]. Like all biomarkers, IMP3 status must be evaluated within the context of histology and clinical presentation. In general, to allow for a more confident distinction between reactive and dysplastic lesions, a panel of markers is often essential; for example, IHC analysis of CK20, p53, and CD44 improves the diagnostic accuracy of urinary bladder cancer detection and has been included in workups or urinary biopsies (Figure 3, Table 1).

Novel promising biomarkers for differentiating benign from malignant mesothelial lesions include enhancer of zeste homolog 2 (EZH2) and 5-hydroxymethylcytosine (5hmC) [207]. Increased nuclear EZH2, along with the loss of nuclear 5hmC immunostaining, favors malignant mesothelioma over reactive mesothelial lesions; however, this requires further validation [207]. Bcl-2-associated athanogene 3 (BAG3), a protein involved in the stress response, is a promising biomarker for cervical intraepithelial neoplasia [208]. A benign squamous epithelium is negative for BAG3 immunostaining, whereas all precancerous lesions display cytoplasmic/nuclear BAG3 immunostaining, which is significantly associated with the grading of intraepithelial dysplasia [208]. The expression of BAG3 has also been documented in ovarian and endometrial carcinomas, highlighting its potential use in the gynecological field [209]. However, BAG3 remains to be established for routine diagnostic practice.

## 4. Future Perspectives

To develop new and better biomarkers for routine practice, it is necessary to identify the underlying mechanisms of inflammation-associated carcinogenesis.

**Figure 4:**
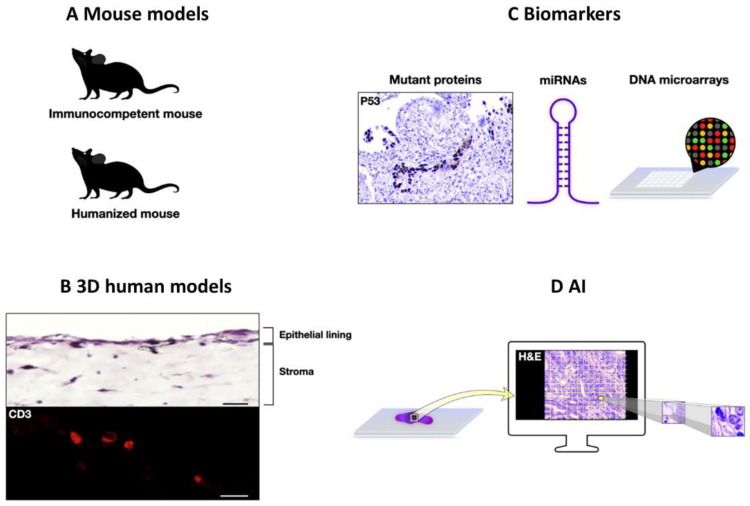
Future perspectives in differential diagnosis between reactive non-neoplastic and neoplastic lesions. (**A**) Immunocompetent mouse models including gain or loss of gene function complemented with humanized mouse models could improve our understanding of human inflammatory-associated diseases. (**B**) Three-dimensional human models providing a mechanistic insight into the tissue response to chronic inflammatory stimuli: representative hematoxylin and eosin (H&E) staining and immunofluorescent micrographs of a three-dimensional human colonic immunocompetent model with embedding of CD45+ cells. The localization of CD3+ lymphocytes in the organotypic 3D model was assessed by immunofluorescence, using an antibody specific for CD3 (red). Nuclei were counterstained with DAPI. Scale bar: 50 μm (upper photo); 25 μm (lower photo). (A. Bergonzini and T. Frisan, personal communication) (**C**) Novel biomarkers: incorporation of mutant-specific antibodies like against p53 hotspot mutants, as well as miRNAs, and DNA microarray applications could improve diagnostic accuracy. (**D**) Artificial intelligence (AI)-based prediction models analyzing routine histopathological H&E-stained sections.

Research models are important tools for obtaining insights into inflammation, tissue response, and cancer. Despite challenges in recapitulating complex human pathology, rodents, such as mammals, share several anatomical and physiological similarities with humans, providing dynamic research models to assess histopathological alterations and define underlying molecular mechanisms (Figure 4A) [210]. Genetically engineered immunocompetent mouse models with loss or gain of gene function have substantially contributed to the study of intestinal mucosal responses in IBD [40,199], gastric metaplasia, and ADM [61,63]. To better model the human immune response, the employment of humanized mice, in which immunodeficient mice are engrafted with human hematopoietic cells, improves our understanding of human inflammatory signaling pathways [211]. Recently, Flavell and colleagues generated a humanized mouse model that enabled the presence of human neutrophils in the mouse blood periphery for the first time [212] (Figure 4A).

The advancement of 3D models is growing exponentially, bridging the gap between traditional 2D monolayer cultures and complex animal models [213]. Three-dimensional assays have been employed to study the esophageal response to inflammation [214], pancreatic ADM [215], IBD [216], and colorectal cancer carcinogenesis [217]. In addition, the development of immunocompetent 3D mucosal models that recapitulate the colonic mucosa offers a unique opportunity to study different immunological scenarios mimicking human physiology and pathology (Figure 4B). Similarly, human organ on-a-chip models recapitulating human organs allow for the study of disease development [218].

In situ assays, including IHC, currently play a central role in pathology [219]. The introduction of R132H mutation-specific isocitrate dehydrogenase 1 (IDH1) for the differential diagnosis of astrocytoma from astrocytosis has brought about a revolution in pathology (Table 1) [107]. Along this line, the generation of antibodies against p53 hotspot mutants tested in paraffin-embedded tumors highlights their potential applications in routine immunostaining (Figure 4C) [220]. Currently, the commonly used p53 antibodies, DO-1 and DO-7, detect both wild-type and mutant p53 [221]; hence, the introduction of p53-mutant specific antibodies could improve diagnostic accuracy in differentiating reactive from dysplastic lesions. Additionally, miRNA tissue expression could be exploited in routine practice for the differential diagnosis of reactive and dysplastic lesions, as miRNAs are stable, allowing their examination in archival material (Figure 4C). Indeed, several studies have revealed that miRNAs can aid in the differential diagnosis between benign and malignant entities [222,223,224,225]. Furthermore, miRNAs are candidate clinical biomarkers in patients with IBD [226]. DNA microarrays, as tools to study gene expression signatures and genotyping, are promising for pathology research and practice (Figure 4C). DNA microarray analysis revealed that *C15orf48* and *KRT9* have distinct expression profiles in PHE and SCC, allowing an accurate distinction between these two entities [227]. Of course, the introduction of array-based applications in routine practice is challenging because evaluation and validation are not straightforward.

Recent advances in the field of digital pathology, facilitated by the use of state-of-the-art slide scanners, broadband internet connection, and enhanced storage capacity, are expected to significantly improve pathological diagnosis and provide vital information related to prognosis and therapy. With digital pathology, it is now possible to apply artificial intelligence (AI) algorithms in both clinical and research settings [228,229]. As AI is becoming increasingly capable, features extracted from whole-slide digital pathology images could reveal novel aspects of tissue that complement the visual inspection of hematoxylin and eosin (H&E) sections. Deep learning based on convolutional neural networks fragments histopathological sections, allowing classification based on different morphological patterns (Figure 4D) [230]. Successful AI models have enabled the prediction of microsatellite instability in solid cancers [231], as well as histological and molecular subtyping in non-small-cell lung carcinomas [232] and endometrial cancer [233]. Deep learning models enable the association of histological H&E images, including healthy and pathological tissues, with gene expression status, allowing the study of how gene expression shapes tissue morphology [234]. Notably, the implementation of AI incorporating collagen-based features could differentiate CP from PDAC with 91.3% accuracy [235], suggesting that AI can aid in such histopathological challenges (Figure 4D). Furthermore, AI could help us decipher the interactions among the cells of the tumor microenvironment, in addition to accurately predicting the presence of specific molecular alterations and response to various cancer immunotherapies [229].

The role of the microbiome in tissue homeostasis is highly appreciated, emphasizing the necessity of incorporating microbes into experimental design. We have demonstrated the immunomodulatory role of genotoxigenic *Salmonella* in the mouse intestine, stressing the complex crosstalk between the microbiome and intestinal homeostasis [236,237]. Recently, D. Hanahan incorporated the term “polymorphic microbiomes” as an enabling characteristic, highlighting the role of the microbiome in the acquisition of cancer hallmarks [238]. Despite advances in understanding the role of the microbiome, we are clearly at the beginning of capturing the host–microbiome interplay.

## 5. Conclusions

Differentiating reactive atypia from true dysplasia is challenging, as non-neoplastic epithelial lesions often exhibit significant cytological and architectural atypia that can be accompanied by dense fibrosis, often making it impossible to render a definite diagnosis. Morphological evaluation remains in the A-to-Z towards diagnosis. Immunohistochemistry can be employed as an adjunct to distinguish reactive lesions from dysplasia, although the results are often inconclusive. In this review, we described the morphological alterations along with the underlying mechanisms involved in tissue response during persistent inflammation, focusing on the digestive system, and provided an update of tissue-based biomarkers that could help in such diagnostic dilemmas. Ongoing advances in molecular biology and AI are expected to yield novel biomarkers that will complement visual inspection and facilitate optimal pathological diagnosis.

We are beginning to understand the precise molecular and cellular events that shape tissue changes during persistent inflammation. Future perspectives point to promising avenues for research and clinical interventions that could allow the introduction of novel tissue-based biomarkers that will improve treatment decisions and ultimately benefit patient health care. 

## Figures and Tables

**Table 1:** Differentiating reactive from true dysplastic lesions employing immunohistochemistry (IHC). Immunostaining pattern of tissue-based protein biomarkers in reactive and neoplastic lesions. (−): immunonegativity; (+): immunopositivity. Abbreviations: ACA: adenocarcinoma; AHNAK2: Protein AHNAK2; AMACR(P504s): alpha-methylacyl-CoA racemase; BAP1: BRCA1-Associated Protein-1; BillN: biliary intraepithelial neoplasia; CK-1ε: Casein Kinase 1ε; CIS: carcinoma in situ; CNS: central nervous system; CK17: Cytokeratin 17; CK20: Cytokeratin 20; CITED1: Glu/Asp-rich carboxy-terminal domain, 1; COX2: cyclooxygenase 2; CRC: colorectal carcinoma; DEC1: differentiated embryonic chondrocyte gene 1; ECC: extrahepatic cholangiocarcinoma; EHBDCa: carcinoma of the extrahepatic bile duct; EMA: Epithelial Membrane Antigen; FN1: Fibronectin-1; GBC: gallbladder carcinoma; FC: follicular carcinoma; FVPC: follicular variant of papillary carcinoma; HBME-1: Hector Battifora mesothelial–1; HGD: high-grade dysplasia; HMGA1/2: High-mobility group containing AT-hook; HSIL: high-grade squamous intraepithelial lesion; HGUC: high-grade urothelial carcinoma; ICC: intrahepatic cholangiocarcinoma; IND: indefinite for dysplasia; IDH: isocitrate dehydrogenase; IMP3 Insulin-like growth factor II messenger ribonucleic acid (mRNA)-binding protein 3; IND: indefinite for dysplasia; Lewis(y) antigen: blood group 8, BG8; LGD: low-grade dysplasia; LSIL: low-grade squamous intraepithelial lesion; MMP-1: matrix metalloproteinase 1; MTAP: methylthioadenosine phosphorylase; PCNA: proliferating cell nuclear antigen; PDAC: pancreatic ductal adenocarcinoma; Pdx1: Pancreatic progenitor and duodenal homeobox 1; PTC: papillary thyroid carcinoma; RA: reactive atypia; RC: metaplastic cervical squamous epithelium with reactive changes; RUA: reactive urothelial atypia; SMAD4: SMAD family member 4; SOX2: SRY-box 2; TERT: human telomerase reverse transcriptase (TERT); VHL: von Hippel–Lindau; VIN: vulvar intraepithelial neoplasia.

Immunostaining Pattern				
Anatomical Position	Protein (s)	Reactive Lesions	Precancerous–Cancerous Lesions	Reference
CNS	**EGFR**	**Gliosis**: (+) weak membranous	**Gliomas**: (+) strong membranous	[105,106]
	**IDH1** **p.R132H**	**Gliosis**: (−)	**Gliomas**: usually (+) diffused and strong cytoplasmic and weak nuclear	[107,108,109]
	**P53**	**Gliosis**: (−)	**Gliomas**: occasionally (+) diffused and strong nuclear	[107,108,109]
Oral cavity	**CK-1ε**	**Atypical squamous epithelium**: (+) weak nuclear	**Carcinoma in situ**: (+) strong nuclear	[110]
	**CD44**	**Atypical squamous epithelium**: (+) weak membranous	**Carcinoma in situ**: (+) strong membranous	[110]
	**E-Cadherin**	**Pseudoepitheliomatous hyperplasia**: (+) membranous	**Squamous cell carcinoma**: decreased (+) membranous in the invasive front	[111]
	**DEC1**	**Atypical squamous epithelium**: (+) strong nuclear	**Carcinoma in situ**: (+) weak nuclear	[110]
	**Ki67**	**Pseudoepitheliomatous hyperplasia**: (+) nuclear restricted in basal and parabasal cells	**Dysplasia**: often (+) extended to the spinous layer	[191]
	**MMP-1**	**Pseudoepitheliomatous hyperplasia**: (+) cytoplasmic with a basal cell pattern	**Squamous cell carcinoma**: (+) diffused cytoplasmic	[19]
	**PCNA**	**Inflammatory lesion**: (+) nuclear in the basal layer	**Dysplasia**: consistently (+) nuclear in the suprabasal layer	[112,113]
	**P16^INK4a^**	**Inflammatory lesion**: (−) or minimal (+) cytoplasmic/nuclear restricted in the basal cells	**Dysplasia**: (−) or often (+) strong and diffused cytoplasmic/nuclear in the middle and upper thirds or (−)	[112,194]
	**P53**	**Pseudoepitheliomatosis hyperplasia**: occasionally (+) moderate-intensity nuclear with a basal-cell layer pattern	**Dysplasia/squamous cell carcinoma**: often (+) intense and diffused nuclear	[19,111,191]
Esophagus	**Ki67**	**Normal/RA**: focal (+) nuclear, usually restricted to the lower third	**HGD/carcinoma**: (+) diffused nuclear	[114]
	**P53**	**Normal/RA**: usually (−) and to a lesser extent focal (+) weak nuclear	**HGD/carcinoma**: (+) diffused and intense nuclear and rarely (−) (null pattern)	[114]
Esophagus (Barrett’s)	**AMACR (P504S)**	**IND**: usually (−) and to a lesser extent (+) with focal cytoplasmic	**LGD**: often (+) diffused and, to a lesser extent, focal cytoplasmic; **HGD/ACC**: usually (+) diffused and, to a lesser extent, focal cytoplasmic	[115,116,117]
	**IMP3**	**IND**: rarely (+) with cytoplasmic and membranous	**LGD**: occasionally (+) with cytoplasmic and membranous; **HGD**: often (+) with cytoplasmic and membranous	[118,192,193]
	**Ki67**	**BE**: (+) nuclear at the base of the crypt	**Dysplasia**: (+) diffused nuclear	[119]
	**P53**	**BE**: (−)	**LGD**: usually (+) diffused nuclear; **HGD**: regularly (+) diffused nuclear	[120,190]
Stomach	**AMACR** **(P504S)**	**Non-neoplastic epithelium**: (−) and rarely (+) weak cytoplasmic	**Dysplasia/adenocarcinoma**: usually (+) moderate and strong cytoplasmic	[121]
	**Ki67**	**RA**: (+) nuclear with a limited expression pattern	**LGD/HGD**: (+) often diffused nuclear (with an expansion of the proliferating zone)	[122,123]
	**IMP3**	**RA**: often (+) with focal cytoplasmic and membranous (in the basal part of the cell)	**LGD**: often (+) weak cytoplasmic and membranous; **HGD**: often (+) diffused moderate/intense cytoplasmic and membranous	[124]
	**P53**	**RA**: (−) or (+) focal and rarely diffused nuclear	**LGD**: rarely (+) weak-to-moderate nuclear; **HGD**: often (+) moderate/strong nuclear	[122,123]
Colon	**AMACR (P504S)**	**IND**: rarely (+) focal cytoplasmic	**LGD/HGD/ACC**: (+) often diffused cytoplasmic	[115]
	**P21^WAF1^**	**Regenerative atypia and indefinite for dysplasia**: (+) strong nuclear mainly located in the superficial portion of colonic glands that are p53 (−)	**Dysplasia and ACA**: (−) in areas with (+) diffused P53 status	[125,126,127]
	**P53**	**Regenerative atypia and indefinite for dysplasia**: (+) mainly few isolated cells with weak and moderate and to a lesser extent basal/nested nuclear	**Dysplasia and ACA**: (+) strong and diffused, basal/nested, and to a lesser extent few isolated cells nuclear	[125,126,127]
Biliary tract	**CD10**	**Normal/RA**: (+) strong membranous with continuous apical pattern	**HGD/ECC**: (−) and rarely (+) focal moderate membranous	[128]
	**CD24**	**Normal/RA**: (−) or (+) focally membranous/cytoplasmic	**Dysplastic epithelium/ECC/ICC/GBC**: (+) strong membranous/cytoplasmic	[129]
	**P-Cadherin**	**Normal/RA**: (−) or rarely (+) focal membranous	**Dysplastic epithelium/ECC/ICC/GBC**: often (+) focal/diffused membranous	[129]
	**HMGA1, HMGA2**	**RA**: (+) weak/moderate nuclear	**Carcinoma**: (+) intense nuclear	[130]
	**Mesothelin**	**RA**: (−)	**High-grade BillN and EHBDCa**: often (+) diffused cytoplasmic and membranous	[131]
	**P53**	**Normal/RA**: (−) or (+) focal weak/moderate nuclear	**Dysplastic epithelium/ECC/ICC/GBC**: often (+) diffused and intense nuclear	[129,130]
	**S100A, S100A4**	**Normal/RA**: (−) or rare (+) cytoplasmic and nuclear	**Dysplasia (including high-grade BillN)/carcinomas arising in periampullary duodenal mucosa/EHBD**: usually (+) diffused membranous and cytoplasmic	[131,132]
	**S100P**	**RA**: (−) or rarely (+) nuclear, weak cytoplasmic	**High-grade BillN and ICC**: occasionally (+) diffused and intense nuclear and cytoplasmic	[133,134]
Gallbladder	**P16^INK4a^**	**Normal/RA**: (−) and rarely (+) nuclear	**Dysplasia/carcinoma**: often (+) diffused and intense nuclear	[135]
	**P53**	**Normal**: (−)	**Dysplasia/carcinoma**: often (+) diffused and intense nuclear	[195]
	**COX2**	**Normal**: (−) and rarely (+)	**Dysplasia/carcinoma**: often (+) diffused cytoplasmic/nuclear	[195]
Pancreas	**DPC4 (SMAD4)**	**Benign**: (+) diffused cytoplasmic and occasionally nuclear	**PDAC**: usually (−), occasionally (+) diffused cytoplasmic and nuclear	[136]
	**IMP3**	**Normal/pancreatitis**: (−) and rarely (+) focal membranous and cytoplasmic	**PDAC**: usually (+) diffused membranous and cytoplasmic	[137,138,139,140,141,142]
	**Maspin**	**Normal**: usually (−), rarely (+) focal nuclear and cytoplasmic	**PDAC**: usually (+) diffused nuclear and cytoplasmic	[142]
	**Mesothelin**	**Pancreatitis**: (−) and rarely (+) with focal membranous and cytoplasmic	**PDAC**: usually (+) diffused membranous and cytoplasmic	[140]
	**P53**	**Pancreatitis**: (−), rarely (+)	**PDAC**: often (+) diffused intense nuclear	[138,139]
	**S100A4**	**Normal**: (+) focal membranous and cytoplasmic	**PDAC**: often (+) diffused membranous and cytoplasmic	[141]
	**S100P**	**Normal**: usually (−), rarely (+) focal nuclear and cytoplasmic	**PDAC**: usually (+) diffused nuclear and cytoplasmic	[142]
	**VHL**	**Normal**: (+) diffused cytoplasmic	**PDAC**: (+) focal cytoplasmic	[141,142]
Urinary Bladder	**AHNAK2**	**RUA**: (−)	**Urothelial CIS**: (+) diffused cytoplasmic	[143]
	**AMACR (P504s)**	**RUA**: (−)	**Urothelial CIS**: often (+) diffused and intense cytoplasmic	[144,145]
	**CD44**	**RUA**: usually (+) membranous with a basal-to-full-thickness pattern	**Urothelial CIS**: often (−) or (+) focal membranous with a basal pattern	[146,147,148]
	**CK5/6**	**RUA**: (+) diffused and intense membranous with full-thickness pattern	**Urothelial CIS**: (−) and rarely (+) membranous with a basal pattern	[149]
	**CK20**	**RUA**: (+) membranous limited to umbrella cells	**Urothelial CIS**: usually (+) full-thickness membranous	[144,145,146,147,148,150,151,152,153,154]
	**HER2/Neu**	**RUA**: usually (−) or (+) faint membranous limited to umbrella cells	**Urothelial CIS**: often (+) moderate-to-intense full-thickness membranous	[151,152,155]
	**Lewis(y) antigen**	**RUA**: (+) patchy membranous	**Urothelial CIS**: (+) intense full-thickness membranous	[155]
	**P16^INK4a^**	**RUA**: occasionally (−) or (+) weak nuclear and cytoplasmic	**Urothelial CIS/HGUC**: (+) diffused and intense nuclear and cytoplasmic	[156]
	**P53**	**RUA**: (−) or (+) patchy and weak nuclear	**Urothelial CIS**:often (+) diffused and intense nuclear or rarely (−)	[148,150,151,152,153]
Uterine Cervix	**Cyclin E**	**RC and atrophic cervical epithelium**: mainly (−), rarely (+) nuclear	**HSIL**: occasionally (+) diffused full-thickness nuclear	[157,158,159]
	**IMP3**	**Normal**: (−); **tubular metaplasia**: (−)	**In situ adenocarcinoma**: (+) diffused and intense nuclear and cytoplasmic	[160]
	**Ki67**	**RC and atrophic cervical epithelium**: few (+) scattered basal and parabasal nuclei, rarely (+) in the upper two thirds	**HSIL**: (+) diffuse full-thickness nuclear	[157,158,159,161,162,163,164,165]
	**P16^INK4a^**	**Normal, RC, and atrophic cervical**: mainly (−) and occasionally (+) weak, in the lower half of the epithelium nuclear and cytoplasmic	**LSIL**: (+) varying intensity, mainly in the lower half of the epithelium nuclear and cytoplasmic; **HSIL**: (+) diffuse and intense full-thickness nuclear and cytoplasmic	[157,158,159,160,166,167,168,169]
	**P53**	**Atypical tubal metaplasia**: (−) and often focal weak (+)	**Uterine serous carcinoma**: frequently (+) diffused and moderate-to-intense nuclear; rarely moderate (+) nuclear or (−)	[163,164,165]
	**TERT**	**Atypical tubal metaplasia**: (−)	**Uterine serous carcinoma**: (+) weak, moderate, and intense nuclear	[163,164,165]
Vulva	**CK17**	**Normal/reactive entity**: usually (−); to a lesser extent (+) patchy and weak suprabasal and rarely (+) moderate–intense suprabasal membranous	**VIN**: usually (+) moderate–strong full-thickness or suprabasal membranous and, to a lesser extent, patchy moderate–intense suprabasal membranous	[170,171]
	**P53**	**Reactive entity**: (+) patchy and weak nuclear	**VIN**: often (+) diffused and intense nuclear	[170,171]
	**SOX2**	**Normal/lichen sclerosus**: usually (+) scattered faint or moderate/intense basal and suprabasal nuclear	**VIN**: usually (+) moderate/intense and full-thickness nuclear	[172]
Pleura	**BAP1**	**Reactive mesothelial hyperplasia**: (+) diffused nuclear	**Malignant mesothelioma**: frequent (−)	[173,174,175,176,177,178]
	**Desmin**	**Reactive mesothelial hyperplasia**: usually intense and diffused (+) cytoplasmic	**Malignant mesothelioma**: usually (−), occasionally focal, and rarely diffused (+) cytoplasmic with faint/moderate intensity	[179,180]
	**EMA**	**Reactive mesothelial hyperplasia**: usually (−), occasionally (+) focal membranous, and rarely (+) diffused membranous	**Malignant mesothelioma**: usually (+) intense and diffused membranous	[179,180]
	**MTAP**	**Reactive mesothelial hyperplasia**: (+) diffused cytoplasmic	**Malignant mesothelioma**: frequent (−)	[173,174,175,176,177,178]
	**P53**	**Reactive mesothelial hyperplasia**: usually (−) and rarely (+) intense nuclear	**Malignant mesothelioma**: often diffused and intense (+) nuclear	[179,180]
Thyroid gland	**BRAF p.V600E**	**Normal**: (−)	**PTC**: (+) diffused cytoplasmic	[181,182]
	**CITED1**	**Normal/RA**: (−)	**PTC**: (+) diffused cytoplasmic and nuclear	[181,182,183,184,185,186,187,188]
	**CK19**	**Normal/RA**: mainly (−) and to a lesser extent (+) focal weak/moderate membranous	**PTC**: frequently (+) moderate/intense membranous	
	**CD56**	**Normal/RA**: (+) intense membranous	**PTC**: mainly (–) and, to a lesser extent, (+) weak membranous	
	**FN1**	**Normal/RA**: (−)	**PTC**: (+) cytoplasmic and membranous	
	**Galectin-3**	**Normal/RA**: (−)	**PTC**: frequently (+) diffused cytoplasmic	
	**HBME-1**	**Normal/RA**: (−)	**PTC**: frequently (+) diffuse and intense membranous	
	**IMP3**	**Thyroiditis Hashimoto**: (−)	**FVPC, FC**: often (+) with diffused membranous and cytoplasmic	[189]

## Data Availability

Not applicable.

## Abbreviations

ACA: adenocarcinoma; ADM: acinar-to-ductal metaplasia; AHNAK2: Protein AHNAK2; AID: activation-induced cytidine deaminase; AMACR(P504s): alpha-methylacyl-CoA racemase; AI: artificial intelligence; ATF3: activating transcription factor 3; BAG3: Bcl-2-associated athanogene 3; BAP1: BRCA1-Associated Protein-1; BCL-10: B-cell lymphoma/leukemia 10; BE: Barrett’s esophagus; BHA15: Basic Helix–Loop–Helix Family Member A15 (widely known as MIST1); BillN: biliary intraepithelial neoplasia; BME-1: Hector Battifora mesothelial–1; CAC: colitis-associated cancer; CD44v9: CD44 variant 9; CDKN2A: cyclin-dependent kinase inhibitor 2A; CDX1: Caudal-type homeobox 1; CDX2: Caudal-type homeobox 2; CK-1ε: Casein Kinase 1ε; CK19: Cytokeratin 19 CC: Crohn’s colitis; CIS: carcinoma in situ; CNS: central nervous system; CK17: Cytokeratin 17; CK20: Cytokeratin 20; CITED1: Glu/Asp-rich carboxy-terminal domain, 1; COX2: cyclooxygenase-2, CP: chronic pancreatis; CRC: colorectal carcinoma; CRP: C-reactive protein, DEC1: differentiated embryonic chondrocyte gene 1; DNMT-1: DNA methyltransferase-1; DPC4: Deleted in Pancreatic Cancer 4; ECC: extrahepatic cholangiocarcinoma; EGF: epidermal growth factor; EGFR: epidermal growth factor receptor; ECM: extracellular cell matrix; EGFR: epidermal growth factor receptor; EHBD: extrahepatic bile duct; EMA: Epithelial Membrane Antigen; ERK: extracellular signal-regulated kinase; EZH2: Enhancer of zeste homolog 2; FAP: fibroblast activation protein; FC: follicular carcinoma; FVPC: follicular variant of papillary carcinoma; GFAP: Glial fibrillary acidic protein; H&E: hematoxylin and eosin; FGF7: fibroblast growth factor 7; FN1: Fibronectin-1; GBC: gallbladder carcinoma; HGD: high-grade dysplasia; HMGA1/2: High-mobility group containing AT-hook; HSIL: high-grade squamous intraepithelial lesion; HGUC: high-grade urothelial carcinoma; IBD: inflammatory bowel disease; ICC: intrahepatic cholangiocarcinoma; Ihh: Indian hedgehog; IND: indefinite for dysplasia; INFγ: interferon γ; IDH: isocitrate dehydrogenase; IL-13: Interleukin 13; IMP3: Insulin-like growth factor II messenger ribonucleic acid (mRNA)-binding protein 3; IND: indefinite for dysplasia; JNK: c-jun N-terminal kinase; Lewis(y) antigen: blood group 8, BG8; LGD: low-grade dysplasia; LGR5: Leucine-rich repeat-containing G protein-coupled receptor 5; LSIL: low-grade squamous intraepithelial lesion; MAPK: mitogen-activated protein kinase; miRNAs: microRNAs; MMP-1: matrix metalloproteinase 1; MTAP: methylthioadenosine phosphorylase; mTORC1: mammalian target of rapamycin complex 1; MUC1: Mucin 1; MUC2: Mucin 2; MUC5AC: Mucin 5AC; MUC6: Mucin 6; NF-κB: Nuclear factor-kappa B; PAI-1: plasminogen activator inhibitor 1; PanIN: pancreatic intraepithelial lesion; PCNA: proliferating cell nuclear antigen; PDAC: pancreatic ductal adenocarcinoma; PEH: pseudoepitheliomatous hyperplasia; PDGF: fibrogenic platelet-derived growth factor; Pdx1: pancreatic and duodenal homeobox 1; PPAR-γ: Peroxisome proliferator-activated receptor-γ; PSCs: pancreatic stellate cells; PTC: papillary thyroid carcinoma; PTF1α: pancreas transcription factor 1 subunit α; RA: reactive atypia; RC: metaplastic cervical squamous epithelium with reactive changes; RONS: reactive oxygen and nitrogen species; RUA: reactive urothelial atypia; SCC: squamous cell carcinoma; αSMA: α smooth muscle antigen; SMAD4: SMAD family member 4; Shh: Sonic hedgehog; SOX2: SRY-box 2; SOX9: SRY-box 9; SPEM: spasmolytic polypeptide-expressing metaplasia; TERT: human telomerase reverse transcriptase; TFF2: trefoil factor family 2; TGFα: transforming growth factor α; TGFβ1: transforming growth factor β1; TIMPs: inhibitors of MMPs; TNFα: Tumor necrosis factor α; UC: ulcerative colitis; VHL: von Hippel–Lindau; VIN: vulvar intraepithelial neoplasia; Wnt: Wingless-related integration site.
